# A process study of early achievements and challenges in countries engaged with the WHO Special Initiative for Mental Health

**DOI:** 10.1186/s13033-024-00652-8

**Published:** 2024-10-21

**Authors:** Alastair Ager, Sabrina Hermosilla, Alison Schafer, Dévora Kestel

**Affiliations:** 1 Institute for Global Health and Development, Queen Margaret University, Edinburgh, Scotland; 2 Department of Population and Family Health, Mailman School of Public Health, Columbia University, New York, USA; 3 Department of Mental Health and Substance Use, World Health Organisation, Geneva, Switzerland

## Abstract

**Background:**

There is increasing awareness of the importance of the transformation of mental health systems. Launched in 2019, the WHO Special Initiative for Mental Health seeks to accelerate access to quality and affordable care for mental health conditions as an integral component of Universal Health Coverage. Nine countries are currently engaged with the initiative.

**Methods:**

This study reviewed processes of implementation—and progress achieved—across all settings by late 2022. It involved review of 158 documents provided by WHO relating to Special Initiative activities and 42 interviews with country-level stakeholders, WHO Regional and HQ personnel engaged with the initiative, and core donors. Documents were thematically coded using a template based upon the WHO framework of health system building blocks. Responses to structured interviews were coded based on an emergent thematic framework.

**Results:**

Documentation reported similar achievements across all domains; however challenges were reported most frequently in relation to service delivery, leadership and governance, and workforce. Issues of financing were notable in being twice as likely to be reported as a challenge than a success. Interviews indicated four major areas of perceived achievement: establishing a platform and profile to address mental health issues; convening a multi-stakeholder, participatory engagement process; new, appropriate services being developed; and key developments in law, policy, or governance around mental health. The planning process followed for the initiative, senior country-level buy-in and the quality of key personnel were the factors considered most influential in driving progress. Ambivalent political commitment and competing priorities were the most frequently cited challenges across all interviewees.

**Conclusions:**

The role of the Special Initiative in raising the profile of mental health on national agendas through a participatory and inclusive process has been widely valued, and there are indications of the beginnings of transformational shifts in mental health services. To secure these benefits, findings suggest three strategic priorities: increasing political prioritisation and funding for systems-level change; clearly articulating sustainable, transformed models of care; and promoting feasible and contextualised measures to support accountability and course correction. All are of potential relevance in informing global strategies for mental health systems transformation in other settings.

**Supplementary Information:**

The online version contains supplementary material available at 10.1186/s13033-024-00652-8.

## Background

The growing recognition of the large proportion of the global burden of disease attributable to mental health conditions [1] has increasingly been accompanied by an acknowledgement of the severe limitations of many health systems to adequately respond to these needs [2–5]. The last decade has seen a number of initiatives targeting the development of mental health systems [4, 6], with notable advance in addressing factors such as community awareness [6], intervention protocols, [7] and human resource capacity [8]. Although many of these developments have been promising, findings have generally pointed to the importance of transformational system-wide investment [9].

Under the World Health Organisation’s target for 1 billion more people enjoying better health and well-being, mental health is a key area of work for accelerated implementation in WHO’s 13th General Program of Work (GPW13), which covers the period 2019–2023 (and has been extended to 2025). The WHO Special Initiative for Mental Health (‘Special Initiative’) was initially established as a five-year programme by the Department of Mental Health and Substance Use (MSD) in 2019.

The Special Initiative aims to advance policies, advocacy, and human rights and to scale-up quality interventions and services for people with mental health conditions, including substance use and neurological disorders. It seeks to support the transformation of mental health systems and services envisioned in the World Mental Health Report 2022 [9] via universal health coverage (UHC) for mental health conditions through access to quality and affordable care. The Special Initiative targets align with those indicated in the Comprehensive Mental Health Action Plan 2013–2030 [10].

Six countries—Bangladesh, Jordan, Paraguay, the Philippines, Ukraine and Zimbabwe—began design and implementation work for the Special Initiative in January 2020 and have been implementing since based on individual country specific work plans. Due to disruptions caused by the COVID-19 pandemic, work in these countries has been extended to the end of 2025. A further two countries, Nepal and Ghana, joined the initiative in late 2021, followed by Argentina in 2022. For each of these countries the initiative will run for a five-year period.

In mid-2022, WHO commissioned a review to collate learnings regarding progress of the initiative, and recommendations to inform the further support WHO will provide to countries as the Special Initiative progresses. Across the nine participating countries—at their different phases of implementation—views on processes and progress were to be sought from Ministries of Health; WHO Country, Regional and Headquarters Offices; donors; and relevant national organisations (including for persons with lived experiences of mental health conditions). This paper presents key findings from this review with the aim of informing wider efforts to secure transformation in national mental health systems worldwide.

## Methods

Data sources comprised 158 documents provided by the WHO Department of Mental Health and Substance Use relating to the Special Initiative activities in each of the 9 participating countries, and 42 interviews with the specified range of involved stakeholders. Interviewers with relevant language fluency—spanning English, Spanish, Arabic, Ukrainian, Bengali, and Hindi—took responsibility for review of documentation related to a particular country and led interviews in that country. Reflecting the commissioned parameters of the review, in each country one Ministry of Health representative (the nominated Special Initiative lead or their alternate), one WHO country office postholder (the appointed technical lead) and one non-governmental stakeholder (service user, service provider or research organisation representative selected on a quota basis) were interviewed. The remaining 15 interviews were completed with WHO Headquarters (HQ) staff (with significant responsibility for Special Initiative activities), WHO Regional staff (senior officers from each WHO region) and representatives of the major Special Initiative donors.

All interviews were conducted virtually and framed with respect to a common interview guide (set of probe questions) addressing perceived successes and challenges faced in the work of the Special Initiative. The interview guide (see Supplementary File A) was developed by the research team, with feedback from the WHO, to highlight not only perceived areas of advance and difficulty but also—by eliciting analysis of concrete events—the factors to which such progress (or lack of it) was attributed. WHO staff were not present during interviews with non-WHO study participants.

Invitations to interview were sent by email together with documentation assuring confidentiality and the right to decline or withdraw from interview at any stage. Acceptance of this invitation was considered consent for participation. To facilitate notetaking, permission was sought from interviewees to record interviews (with recordings destroyed after anonymized notes were completed). Procedures were reviewed and approved by the research ethics review panel of Queen Margaret University.

Key information from documents was collated using an extraction matrix. Documents comprised baseline assessments, planning documents, correspondence, and progress reports. A coding frame for document analysis was developed based on the structure of the WHO health system building blocks [11]. An audit of 10% of coded documents established acceptable reliability of this coding frame. For interviews, the review team evolved a thematic coding structure in an iterative manner through interview review and discussion. One in five interviews was dual coded by independent reviewers using this coding structure, which established acceptable reliability of this coding frame (inter-rater reliability by domain of 0.90). All coding and data analyses were completed by the review team, without inputs from WHO personnel.

The design was not powered for country-by-country analysis. Where relevant, however, findings are disaggregated by country-level interviews (*n* = 27) compared with those from WHO HQ/Regional staff and donors (*n* = 15). Basic statistical analyses were conducted to guide interpretation of frequency data, consistent with a mixed methods approach [12].

## Results

### Reported achievements

Documentation reported achievements across all WHO health system building block domains [11]. Although leadership and governance issues were the most frequently—and financing issues the least frequently— noted in the reviewed documents, there was overall no significant trend for particular domains being over- or under-represented (*Χ*^2^ = 1.66, df = 3, *p* = .646).

**Fig. 1:**
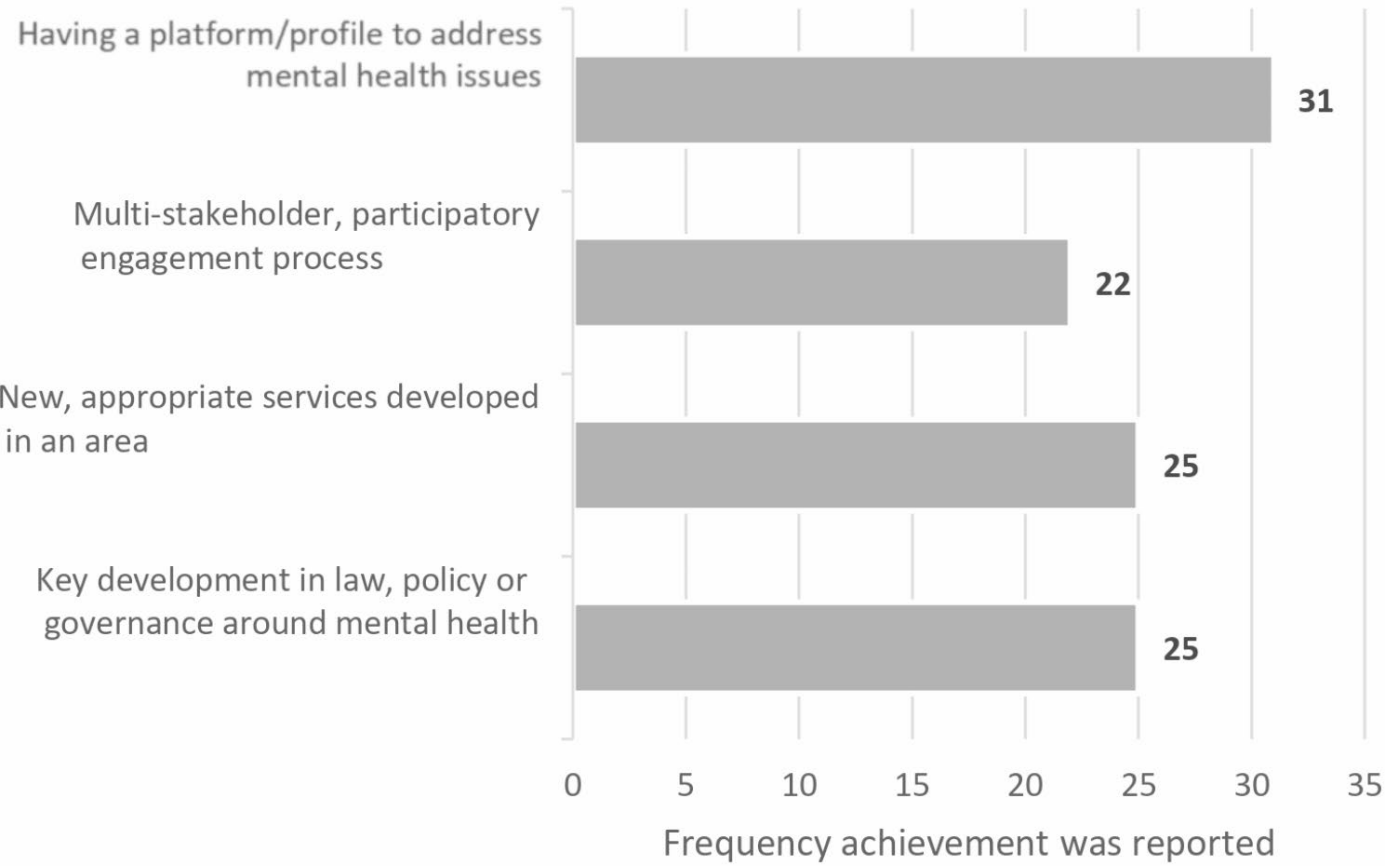
Thematic focus of key achievements (*n* = 103) reported in interviews (*N* = 42)

Building off of the documentation findings, interviews directly addressed interviewees’ understanding of key achievements of the Special Initiative to date. Figure 1 shows the four response categories that emerged from a total of 103 achievements noted in interviews. Overall, there were no significant differences in the pattern of reporting between country-level stakeholders and WHO HQ/Regional staff and donors regarding these categories.

The most noted achievement was that the Special Initiative contributed to establishing a platform and profile to address mental health issues. Whether globally or at country level, the Special Initiative was reported to have increased awareness about mental health and enabled focused dialogue and engagment on this frequently neglected area. This platform served as a stimulus for determining future actions, with training initiatives commonly cited as a priority action. The Special Inititive provided both a platform to convene discussions, as well as a valuable global profile for engagement in the transformation of mental health provision.

*‘Being part of the initiative created a momentum and put mental health on the list of priorities for the national authorities and stakeholders. The start of the initiative prompted work on updating the national action plan which had expired’*, WHO Country Office interviewee.

*‘With our ongoing training programme*,* we plan that half of our workforce at the community level will be trained with basic mental health training in the next three years’*, MoH interviewee.

*‘The initiative has led to awareness creating activities so that the demand for mental health services increases from the community level’*, Non-governmental country-level interviewee.

Convening a multi-stakeholder, participatory engagement process to establish unique country Special Initiative plans over a 5-year period was also commonly viewed as a major achievement, especially amongst country-level stakeholders. A number of interviewees reported on the value of the inclusive process adopted.

*‘This process of revising and developing [the implementation plan] involved the government*,* WHO*,* NGOs*,* and INGOs and integrated everyone in improving service capacity and strengthening systems… this process can inform other national health strategies’*, WHO Country Office interviewee.

*‘Wider stakeholder engagement was good. We would have about 100 participants at key [online] meetings. So*,* we believe this enabled us to develop a specific mental health strategy*,* initiate various activities for services transformation*,* and identify the priorities and implementing strategies for improved provision’*, WHO Country Office interviewee.

*‘The initiative was a catalyst for bringing all the most prominent players—such as government and key other organisations in the mental health field—together and giving them space to harmonise their experiences and expertise’*, Non-governmental country-level stakeholder.

Substantive achievements in service development were also frequently noted, particularly by WHO HQ/Regional staff and donors. New or strengthened services and related resources were highlighted by several interviewees, mentioning for example advances of telehealth or community services development.

*‘We have seen the development of a community mental health intervention… although some of the capacity building around that was pre- Special Initiative*,* I really don’t think it would have scaled to the level—or the quality—it has achieved without it [the Special Initiative]’*, WHO HQ interviewee.

*‘To address availability of medicines*,* there is work with the government to provide a medicines starter kit. Trainees liaise with the mental health coordinators to monitor the utilisation and replenish stores of medication’*, Non-governmental country-level stakeholder.

Key developments in law, policy, or governance around mental health were the final category of achivement widely cited across interviews. Examples included the development of national action plans, the passing of legislation regarding mental health access, or the appointment of a governing board to monitor mental health services.

*‘The Special Initiative complements and supports implementation of our national mental health strategy and action plan’*, MoH interviewee.

*‘Reviewing the Mental Health Act is a potential game changer and will help transform services from the core. If we have updated policies in place, we believe that will change the whole system’*, WHO Country Office interviewee.

*‘There is real pressure from legislators and national advocates to implement the mental health law. That’s one big thing that keeps us moving forward’*, MoH interviewee.

### Factors supporting achievements

**Fig. 2:**
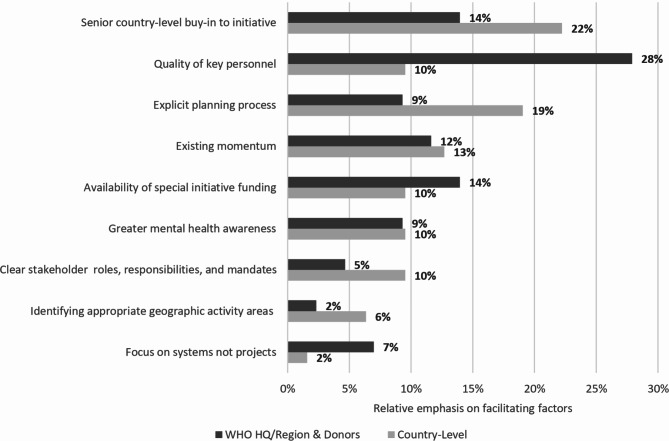
Factors (*n* = 106) to which achievements were attributed in interviews (*N* = 42) (disaggregated by country-level interviews [*N* = 27] cf. WHO HQ/Regional and donor interviews [*N* = 15])

Interviewees identified factors they considered to have facilitated achievements, with major themes shown in Fig. 2. There was broad agreement between interviewees at the country-level and amongst WHO HQ/Regional staff and donors regarding the relevance of these factors. There was no statistically significant difference in the likelihood of their reporting a particular factor as important, other than WHO HQ/Regional staff and donors being statistically significantly more likely to put emphasis on the quality of key personnel (*Χ*^2^ = 12.29, df = 1, *p* < .001).

The factor most frequently referenced by interviewees was senior country-level buy-in to the Special Initiative. While government commitment was a necessary condition of becoming a participating country in the Special Initiative, on-going support from senior leadership regarding factors such as the human rights and primary care orientation of the initiative was crucial to sustain progress.

*‘At the launch meeting in Geneva*,* we had the Secretary General of the Ministry of Health at that time joining in as part of the deliberations and the planning process… I think that helped’*, WHO Regional interviewee.

The quality of key personnel in senior positions in both MoH and WHO country offices was the second most frequently cited factor facilitating progress, with many interviewees—especially (as indicated above) those from WHO HQ/Regional staff and donors. Interviewees stressed these positions as essential to drive progress.

*‘The people at the country offices have been key. Without them and their persistence, the Special Initiative would not have been able to progress. These people were involved in making things work’*, WHO HQ interviewee.

*‘Your local leadership is so important because if you don’t have that*,* you don’t move anywhere*,* no matter how smart you are’*, WHO HQ interviewee.

In line with multi-stakeholder engagement being viewed as a major achievement, many interviewees pointed to the value of the explicit planning process that had been followed for the Special Initiative in their countries. This included reference to specific aspects of the process, such as the use of logframes, kick-off meetings, and consultations. These were all seen to have been helpful to engage a broad range of stakeholders to jointly identify shared objectives.

*‘Planning saw the involvement of many different stakeholders: the Ministry of Health*,* CBOs*,* NGOs*,* schools, etc. Without the WHO, it would not have been possible to gather all these stakeholders together’*, non-governmental country-level stakeholder.

Interviewees saw building on existing momentum in work at the country-level (whether in terms of law, policy, or services) a major benefit for Special Initiative activities:

*‘We tried to position the special initiative not as something which is completely new…but as a continuation of the existing capacity building initiative’*, WHO Regional interviewee.

Other factors seen to facilitate progress included: having clear stakeholder roles, responsibilities, and mandates; greater awareness of mental health issues, whether this was due to the COVID-19 pandemic, humanitarian crises, and, in some cases, identifying appropriate geographical areas for activities where mental health system structures are poorly developed.

*‘The pandemic created an opportunity to highlight the importance of addressing mental health. It improved people’s—including officials’ —awareness of the issue’*, MoH interviewee.

### Reported challenges

Documentation, such as country situational assessments, reported challenges across all health system building block domains, but issues of financing were notable in being twice as likely to be reported as a focus of challenge than of success (*Χ*^2^ = 4.10, df = 1, *p* = .042).

**Fig. 3:**
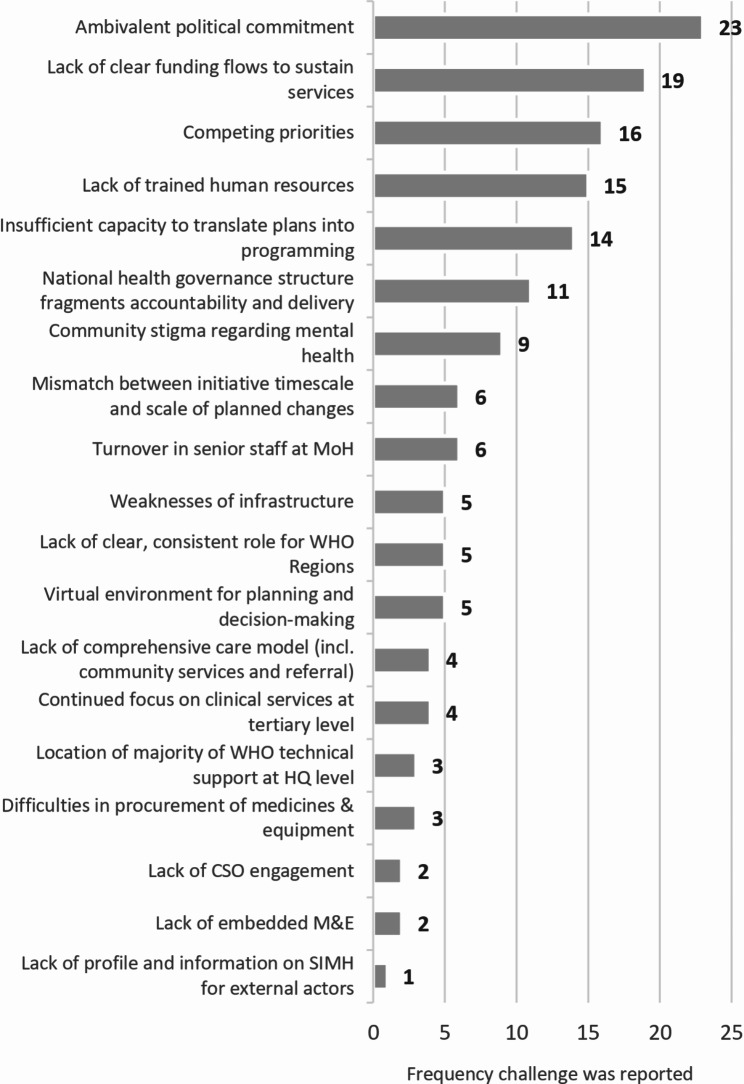
Thematic focus of key challenges (*n* = 153) reported in interviews (*N* = 42)

Major themes identified from interviewees’ discussions about challenges are shown in Fig. 3. Ambivalent political commitment was the most frequently cited issue. This typically referenced uncertainty in follow-through on stated policy objectives. In some instances, this was linked with the wider issue of competing priorities. Although all governments had signed up to the objectives of the Special Initiative, in practice there were a wide range of other government interests and policy areas that were competing for attention and resources.

*‘Challenges arise when it comes to the availability of funds to implement elements of the initiative. It’s very difficult to secure funds from the Ministry of Health. The Ministry is already overwhelmed with the huge needs of the population. Therefore*,* we count on the support of international partners and stakeholders’*, MoH interviewee.

*‘Governments may initially say yes*,* but then as soon as something happens, they focus on the other thing. When other priorities came along, things don’t move forward’*, Donor interviewee.

*‘It would be helpful for top WHO leaders to talk with the senior MoH staff that I report to. This would get MoH more actively involved*,* so that when we implement, we don’t get hiccups. They are often busy and have other areas to focus on*,* and therefore, it is very easy for them to forget about this initiative’*, MoH interviewee.

*‘Upcoming elections provide the potential for distraction and change’*, WHO Country Office interviewee.

Competing priorities were not only seen as a challenge within government, however. For example, many postholders were being faced with multiple tasks beyond mental health, high workload demands on community health workers, and the balance of clinical and supervisory responsibilities of psychiatrists were also mentioned. Many people key to Special Initiative implementation activities were faced with pressure and incentives to engage in other work. This was exacerbated by the COVID-19 pandemic.

*‘During COVID-19*,* we had to change programme plans and develop guidelines to reduce the burden on the frontline workers and maintain their psychosocial well-being’*, MoH interviewee.

The concern over financing and workforce frequently flagged in programme documentation was reinforced in interviews. Regarding mental health financing, the lack of clear and reliable funding flows to sustain services was a frequent focus of discussion, whether the emphasis was on government allocation to mental health provision, the tax basis to enable this, or the perceived continued dependence on donor support.

*‘There are very few psychiatrists and psychologists available*,* and most work in an urban setting. If I want to take mental health services to the rural level*,* the resources I would need for that are still not possible for us to provide’*, MoH interviewee.

*‘Some of the funds came quite late and gave us very little room to utilise these for activities that we had planned’*, MoH interviewee.

*‘We need high-level advocacy for community-based mental health care because still you see some countries tend to prefer to invest in specialised mental health treatments’*, WHO Regional interviewee.

In terms of workforce, the lack of trained human resources was also a frequent theme during interviews, whether addressing the need for recruitment of cadres of personnel, their training and supervision, or the retention of staff within the health system.

*‘It is relatively easy to organise workshop training*,* but you need much more*,* including clinical supervision of trained PHC workers. This is very often neglected*,* and we know very well why it is neglected because it is more challenging to organise: Who is doing that*;* how are they going to do that? With what resources and when? Where is the transport? These kinds of challenges are there’*, WHO Regional interviewee.

Concern over the limited capacity to translate plans into programme implementation was raised by a number of interviewees. It was recognised that the skills and competences required to develop policies, plans, and guidelines were different from those required to drive forward implementation.

*‘I acknowledge the value in having created a group of reform-minded psychiatrists and other mental health professionals*,* but more time is needed for that that group to become to acquire critical mass’*, WHO Regional interviewee.

Many other challenges are noted in Fig. 3. Compared to the relatively focused listing of achievements and the factors facilitating them that emerged from that analysis, interviewees provided a broader range of responses with respect to challenges affecting Special Initiative implementation. The distribution of challenges was significantly different across stakeholder groups (*Χ*^2^ = 58.4742, df = 38, *p* = .018). Lack of clear funding flows was three times more likely to be mentioned by country-level stakeholders and community stigma was only cited by those at country-level. Lack of capacity for translation of plans into implementation was four times more frequently cited in interviews with WHO HQ/Regional staff and donors than with country-level interviewees.

## Discussion

### Factors influencing implementation

The Consolidated Framework for Implementation Research (CFIR) [13] provides a structure for reflecting on these findings. The CFIR identifies constructs (italicized below) relevant to implementation processes across five major domains: the intervention, the setting (both internal and external), individuals involved, and the process.

#### The intervention

The intervention promoted through the Special Initiative can be broadly understood as the establishment of community-based systems for mental health provision, together with the policies, structures, and processes required to sustain access to these. There was wide recognition of the strong *evidence-base* supporting this intervention approach as one likely to maximise access to appropriate and affordable mental health services provision [4]. However, our findings point to two major constraints on progress regarding this intervention approach. First, in a limited number of contexts, there was a remaining commitment to strengthening of tertiary level services, such as specialised psychiatric hospitals or long-stay residential facilities for people with severe mental disorders. This view competed for resources and political commitment in contesting the *relative advantage* of the UHC and primary care focus of the Special Initiative. Second, there was a more prevalent indication of the lack of appreciation of the *complexity* of the full systems-wide requirements of primary mental health care provision. A minority of interviews, for example, showed sharp awareness of the importance of secondary level provision being in place to provide both referral and supervisory systems for provision in primary care and community settings.

#### The setting (internal)

The internal (or ‘inner’) setting for the Special initiative was essentially the partnership between the Ministry of Health and the WHO in each of the implementing countries. It is significant that both key facilitators (‘senior country-level buy-in to initiative’) and barriers (such as ‘turnover in senior staff at MoH’) noted were linked to this domain. Despite differences in the nature and extent of this partnership across settings, in each of the nine implementation settings the core relevance of the *culture* of partnership between WHO and Ministry of Health was clearly recognized. Our findings reinforced the political nature of the initiative in terms of influencing government priorities and resource allocation. *Structural characteristics* of the technical engagement from WHO personnel—especially when episodic and generally remote as is the case of regional or HQ staff—were often not well suited to support such political processes. The ‘ambivalent political commitment’ noted potentially reflected a lack of *readiness for implementation* in terms of a lack of prioritization and resourcing of stated government policy objectives.

#### The setting (external)

The importance of the WHO-MOH partnerships providing the internal setting for the initiative in each country is reinforced when recognising the complexity of the external environment (which the CFIR refers to as the ‘outer setting’) for implementation. This external environment varied widely across the nine implementing countries, but all saw major impacts of *external policy and incentives* related to the COVID-19 pandemic. Although this brought greater awareness of mental health needs, as noted, it severely constrained implementation resulting in delays and re-negotiated timelines. A more positive influence of a shared construct across implementing countries relevant to this domain was the wide recognition that mismatch of *service user needs and resources*—the treatment gap for those presenting with mental health problems—was a powerful motivator for change.

#### Individuals

The role of individuals facilitating implementation is a CFIR domain richly evidenced in the current review. For example, as noted, ‘quality of key personnel’ was amongst the most frequently articulated factors accounting for effective progress amongst WHO Regional officials, Headquarters staff and donors, with respondents regularly highlighting strong *knowledge and beliefs* regarding the intervention and *self-efficacy* underpinning effective leadership.

#### The process

The multi-stakeholder, participatory engagement process of the Special Initiative was frequently cited as a success of the initiative. The participatory nature of these planning and preparatory phases is a major asset for the Initiative going forward, reflecting the important CFIR constructs of *planning* and *engaging*. The concern regarding capacity to translate from policy and planning to programme implementation signals the need for progressive emphasis on process constructs such as *executing* and *reflecting and evaluating*. With regard to the latter, while this review indicates capacity for reflection, the lack of available indicators related to outcomes and impacts at the time of this study highlights the importance of ongoing work to develop and track cross-country logframe indicators related to access, coverage, and human rights.

### Emerging strategic priorities

This study has documented key areas of engagement of the Special Initiative with implementing partners and identified key achievements and challenges in this work to date. The role of the Special Initiative in raising the profile of mental health on national agendas—along with the participatory and inclusive process of planning established to advance the work—is clearly widely valued. There are indications of the beginnings of the transformational shifts in mental health provision envisaged in the 2022 World Mental Health Report [9] across implementing countries.

There are, however, also numerous challenges identified which may constrain progress of the Special Initiative in its proceeding years. Viewing these challenges explicitly in systems terms suggests three particular strategic priorities to secure the planned transformation in implementing countries.

#### Increasing political prioritisation and funding for systems-level change

Whether viewed as an attribute of a successful approach (regarding senior buy-in to the initiative) or as a major challenge (regarding ambivalent political commitment), the issue of political engagement emerged as a prominent concern in documentation and interviews. Systems change is a political, as well as technical, task: varying interests need to be accommodated; budgets need to be re-aligned; competing priorities—as recognised—need to be managed [14]. Explicit attention to the sustainable basis of funding for services is critical given the evidence of the influence of this on treatment coverage across diverse settings [5].

Finally, methods available for more systematic identification of critical bottlenecks and barriers to change, whether via surveys such as those utilised by the WHO-World Mental Health Surveys Initiative [15] or tools associated with approaches such as force field and political economy analysis [16, 17], could be valuable. Participatory methods such as group model building [18, 19] may provide a valuable means to convene stakeholders to collectively address challenges thus identified.

Examples from country settings where momentum has been effectively established suggest there is a key role for regional WHO staff and extended face-to-face engagement by WHO HQ personnel to support such work. Mobilizing champions for change with high public visibility may also reinforce prioritisation. It is crucial to anticipate opportunities for high-level advocacy to ensure conditions for sustainability and to optimally utilise investment case materials, regional meetings, and other appropriate mechanisms for continued activism.

#### Explicitly articulating sustainable, transformed models of care

Although the steps *necessary* to drive greater access to mental health care were consistently documented in planning documents (typically ordered by building blocks), it was not always apparent that these developments in the system would be *sufficient* to deliver greater access to mental health services. For example, while workforce issues were consistently addressed with respect to training initiatives, how training would be built upon in terms of providing supervisory and referral structures, functional ownership or management of these roles, or retention of the mental health workforce, was less frequently articulated.

There are clear risks associated with assumptions that staff trained will be retained and motivated and that secondary level providers will reliably supervise and receive referrals (where appropriate) from primary-level providers. While the Special Initiative appropriately focuses attention on primary level provision, the manner in which secondary services support and facilitate this appears less frequently addressed, despite its importance.

Mapping the health-seeking journeys (i.e., care pathways) of people who seek mental health services may be another useful step to identify weaknesses in the remodelled mental health system in participating countries and contribute towards addressing the necessary changes within the health system [20, 21]. Clear specification of supervision mechanisms and referral and support pathways is warranted in all contexts, with explicit appraisal of risks associated with these not being reliably provided. The governance arrangements required to sustain transformed services (in terms of funding, accountability, employment, conditions of service, etc.) also needs to be more formally articulated across all settings [22]. Significant governance gaps are, indeed, still globally prominent, including inadequate laws, policies and plans, and financial priorities still commonly focusing on psychiatric hospitals [23].

#### Promoting feasible and contextualised measures to support accountability and course correction

Although challenges in monitoring and evaluation were explicitly highlighted only by a minority of interviewees, in systems terms information will continue play a crucial role in addressing many of the challenges noted in Fig. 3. Increasing political prioritisation and financing of systems-level change will be dependent upon information supporting accountability of multiple stakeholders. Indeed, the lack of community-based (and private) mental health services data remains lacking, with most of the global mental health information—and subsequent political decisions—still being based on the narrow scope of data obtained from psychiatric hospitals [24]. More broadly, given the concern regarding capacity to translate from policy and planning to programme implementation, it will be important to track output and outcome indicators in each setting to allow for ‘course correction’ within the funding period.

Implementation needs to be responsive to emerging issues, reflect learning and demonstrate adaptation. Sharing progress indicators in an open and transparent manner—within and across settings—is key in enabling this. Tracking data in relation to the Special Initiative cross-country Indicators on access, coverage, and human rights should be of especial value [25, 26], acknowledging that measures informing these indicators need to be feasible within available data collection capacities and reflect discrete national contexts.

### Limitations

This process review of the WHO Special Initiative for Mental Health has clear limitations for consideration. The review comprises qualitative data, literature analyses, and a contained methodology—not supplemented by quantitative outputs per country, which were unavailable to the time of the study (i.e., assessments of increased access, coverage, or human rights). Further, the modest number of interviewees per country and acknowledgement that each country was self-paced in their progress did not facilitate detailed country-by-country analysis or comparisons. Also, limited interview time and their lack of interviewee engagement in their analysis may have reduced important critical reflections of the findings. Notwithstanding these methodological constraints, the results allowed for overall impressions and patterns to emerge, and some well-defined achievements, challenges, and recommendations for ongoing work in the Initiative were perceptible.

Another important limitation to this review and paper is the intrinsic engagement of WHO and WHO Special Initiative for Mental Health personnel involved. Although it was not feasible to separate WHO from the review process, efforts were made to mitigate prospective bias, including WHO commissioning an independent organisation to complete the review (QMU); WHO not being part of non-WHO interviews or data analyses; and results being summarised independently by QMU researchers. As a qualitative evaluation of a WHO Initiative, the opinions, and interpretations of WHO were as central to the study process as the information provided by non-WHO personnel and programme documentation. Where differences in views arose, these were duly reported and described to form part the overall results (for example, as shown in Fig. 2).

## Conclusions

Evidence regarding early progress of countries engaged in the WHO Special Initiative for Mental Health suggests that participatory and inclusive planning processes have facilitated advancement of national mental health agendas and enabled important policy and service developments towards targeted transformational shifts in mental health services provision. Stakeholders hold somewhat differing views on key challenges to further progress. However, a systems-level perspective suggests key strategic priorities to be increasing political prioritisation and funding for systems-wide change, explicitly articulating sustainable, transformed models of care and promoting feasible and contextualised measures to support accountability and course correction.

## Electronic supplementary material

Below is the link to the electronic supplementary material.

Supplementary Material 1

## Data Availability

Data are available from the second author on request.

## Acronyms

COVID-19: Coronavirus Disease 2019
GPW13: 13th General Program of Work
HQ: Headquarters
MNS conditions: Mental, Neurological and Substance use (MNS) conditions
MoH: Ministry of Health
MSD: Department of Mental Health and Substance Use
NGO: Non-Governmental Organization
PHC: Primary Health Care
UHC: Universal Health Coverage
WHO: World Health Organization

## References

[1] GBD 2019 Mental Disorders Collaborators. Global, regional, and national burden of 12 mental disorders in 204 countries and territories, 1990–2019: a systematic analysis for the global burden of Disease Study 2019. Lancet Psychiatry. 2022;9(2):137–50. 10.1016/S2215-0366(21)00395-3. 35026139 10.1016/S2215-0366(21)00395-3 PMC8776563

[2] Alonso J, et al. Treatment gap for anxiety disorders is global: results of the World Mental Health Surveys in 21 countries. Depress Anxiety. 2018;35(3):195–208. 29356216 10.1002/da.22711 PMC6008788

[3] Thornicroft G, et al. Undertreatment of people with major depressive disorder in 21 countries. Br J Psychiatry. 2017; 210(2):119–24. 10.1192/bjp.bp.116.188078 PMC5288082 27908899

[4] Lancet Commission on Global Mental Health & Sustainable Development. The Lancet, 392, 10157, 1553-1598 (2018). https://www.thelancet.com/commissions/global-mental-health 10.1016/S0140-6736(18)31612-X 30314863

[5] Vigo DV, Kazdin AE, Sampson NA, et al. Determinants of effective treatment coverage for major depressive disorder in the WHO World Mental Health Surveys. Int J Ment Health Syst. 2022;16:29. 10.1186/s13033-022-00539-6. 35739598 10.1186/s13033-022-00539-6 PMC9219212

[6] Lancet Commission on Ending Stigma and Discrimination in Mental Health. Lancet. 2022;400:1438–80. 10.1016/S0140-6736(22)01470-2. 36223799 10.1016/S0140-6736(22)01470-2

[7] Dua T, Barbui C, Clark N, Fleischmann A, Poznyak V, van Ommeren M, et al. Evidence-based guidelines for mental, neurological, and substance use disorders in low- and middle-income countries: summary of WHO recommendations. PloS Med. 2011;8:e1001122. 22110406 10.1371/journal.pmed.1001122 PMC3217030

[8] Keynejad RC, Dua T, Barbui C, et al. WHO Mental Health Gap Action Programme (mhGAP) intervention guide: a systematic review of evidence from low and middle-income countries. BMJ Ment Health. 2018;21:30–4. 10.1136/eb-2017-102750 PMC10283403 28903977

[9] WHO. (2022) World Mental Health Report: Transforming Mental Health for All. https://www.who.int/publications/i/item/9789240049338

[10] World Health Organization. Comprehensive mental health action plan 2013–2030. Geneva: World Health Organization; 2021. Licence: CC BY-NC-SA 3.0 IGO.

[11] World Health Organization. Everybody’s business: strengthening health systems to improve health outcomes. WHO’s Framework for Action. Geneva: World Health Organization; 2007.

[12] Fakis A, Hilliam R, Stoneley H, Townend M. Quantitative analysis of qualitative information from interviews: a systematic literature review. J Mixed Methods Res. 2014;8(2):139–61. 10.1177/1558689813495111.

[13] The Consolidated Framework for Implementation Research. Technical Assistance for users of the CFIR framework. https://cfirguide.org/.

[14] Iemmi V. Establishing political priority for global mental health: a qualitative policy analysis. Health Policy Plann. 2022;37:8, 1012–24. 10.1093/heapol/czac046. 10.1093/heapol/czac046 PMC9384251 35763373

[15] https://www.hcp.med.harvard.edu/wmh/ftpdir/WMHMethods.pdf

[16] Shafaghat T, Zarachi M, Nasab MHI, et al. Force field analysis of driving and restraining factors affecting the evidence-based decision-making in health systems; comparing two approaches. J Educ Health Promot. 2021;10:419. 10.4103/jehp.jehp_1142_20. 35071625 10.4103/jehp.jehp_1142_20 PMC8719555

[17] Reich M. Political economy analysis for health. Bull World Health Organ. 2019;97(8):514. 10.2471/BLT.19.238311. 31384066 10.2471/BLT.19.238311 PMC6653823

[18] Noubani A, Diaconu K, Ghandour L, et al. A community–based system dynamics approach for understanding factors affecting mental health and health seeking behaviors in Beirut and Beqaa regions of Lebanon. Global Health. 2020;16:28. 10.1186/s12992-020-00556-5. 32228648 10.1186/s12992-020-00556-5 PMC7106684

[19] Bou-Orm IR, Moussallem M, Karam J, et al. Provision of mental health and psychosocial support services to health workers and community members in conflict-affected Northwest Syria: a mixed-methods study. Confl Health. 2023;17:46. 10.1186/s13031-023-00547-4. 37794393 10.1186/s13031-023-00547-4 PMC10548701

[20] Volpe U, Mihai A, Jordanova V, Sartorius N. The pathways to mental healthcare worldwide: a systematic review. Curr Opin Psychiatry. 2015;28(4):299–306. 10.1097/YCO.0000000000000164. PMID: 26001921. 10.1097/YCO.0000000000000164 26001921

[21] Harris MG, Kazdin AE, Munthali RJ, et al. Perceived helpfulness of service sectors used for mental and substance use disorders: findings from the WHO World Mental Health Surveys. Int J Ment Health Syst. 2022;16:6. 10.1186/s13033-022-00516-z. 35093131 10.1186/s13033-022-00516-z PMC8800240

[22] Patel V, Saxena S, Lund C, Kohrt B, et al. Transforming mental health systems globally: principles and policy recommendations. Lancet. 2023;402:10402,656–66. 10.1016/S0140-6736(23)00918-2. 37597892 10.1016/S0140-6736(23)00918-2

[23] Mental Health Atlas. 2020. World Health Organization; Geneva, 2021 https://www.who.int/publications/i/item/9789240036703. Accessed 20 June 2024.

[24] Ryan G, De Silva M, Terver JS, Ochi OP, Eaton J. Information systems for global mental health. Lancet Psychiatry. 2015;2(5):372–3. 26360265 10.1016/S2215-0366(15)00097-8

[25] Vigo D, Haro JP, Hwang I, et al. Toward measuring effective treatment coverage: critical bottlenecks in quality- and user-adjusted coverage for major depressive disorder. Psychol Med. 2020. 10.1017/S0033291720003797. 33077023 10.1017/S0033291720003797 PMC9341444

[26] Poynton-Smith E, Orrell M, Osei A, et al. A quantitative analysis of human rights-related attitude changes towards people with mental health conditions and psychosocial, intellectual, or cognitive disabilities following completion of the WHO QualityRights e-training in Ghana. Int J Ment Health Syst. 2023;17:46. 10.1186/s13033-023-00609-3. 38053116 10.1186/s13033-023-00609-3 PMC10698997

