# Supplementary material for: A process study of early achievements and challenges in countries engaged with the WHO Special Initiative for Mental Health
Source: Int J Ment Health Syst. 2024 Oct 21;18:31. doi: 10.1186/s13033-024-00652-8 (PMC11492481; doi:10.1186/s13033-024-00652-8)
Supplement: Supplementary file 1 — Supplementary Material 1 [file 13033_2024_652_MOESM1_ESM.docx]

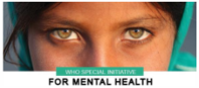


**WHO’s Special Initiative for Mental Health: Mid-term learnings exercise**

**Key Informant Interview Guide**

*Updated 31 October 2022*

Introduction

Thank you for participating in our discussion today. My name is ________________. I am from Queen Margaret University. Thank you for taking the time to speak with me today. As part of the WHO’s Special Initiative for Mental Health Mid-term learning exercise, we are conducting several interviews with stakeholders who are directly involved with this Initiative.

During our discussion I’ll ask questions, let me know if any of these questions are not clear and you need additional clarification. You do not have to answer any questions if you feel uncomfortable. We can skip questions, or stop this discussion at any point. We will use this information to develop recommendations for the Initiative’s continued delivery, your honest feedback is greatly appreciated. Our analysis and report from these interviews will not contain any identifying information about you or your participation.

There are no right or wrong answers to these questions, and the interview should last for around 30 minutes.

Before we begin, I would like to go over a few ground rules:

- I would like to record this session to help with notetaking, are you ok with that? The audio recording and notes will be kept confidential, and recordings will be deleted once notes are completed.

Are you happy to proceed with the interview now?

Do you have any questions before we start?

I will begin recording and taking notes.

**Questions**

1. Based on your experience with the Initiative, what are one or two examples of what you would see as **the greatest successes/achievements** of the Special Initiative in [CONTEXT] since it began [context specific dates especially for NGO/stakeholder]?

Potential probes:

- If just one offered: Can you think of another achievement?
- If more than two offered: We’re happy to focus on a few major achievements in detail at this stage. Which would be the most important, if you had to say.
- If essentially nothing concrete is offered: I noticed in the reports a discussion of [context-specific idea, i.e. human resources], is there anything about that you’d like to discuss?

1. FOR EACH ACHIEVEMENT, *REASONS*: What were the key factors behind this/these successes?

Potential probes:

- What helped make these happen?
- What do you put these successes down to?

1. Now we want to turn to consider **major challenges or problems** related to implementing the Special Initiative. What do you see as the major issues limiting progress of the Initiative in [CONTEXT]?

Potential probes:

Can you tell me more about that?

1. FOR EACH CHALLENGE/PROBLEM: *REASONS:* Why do you think this has been a problem?

Potential probes:

- What are the key factors behind these challenges?
- In what way has this been a challenge?
- Can you give us an example of that?

1. What ***solutions or strategies*** have been adopted to address challenges that you think have been successful?

Potential probes:

Has anything been done to address these challenges to date? How have those activities worked or been implemented?

1. What do you see as the most important **future actions** to secure success for the initiative in [CONTEXT] by 2024/5?

Potential probes:

- Can you tell me more about that?
- What are some potential barriers / challenges to these actions being taken?
- How can those barriers/challenges be overcome?

1. Anything else you would like to share with us today?

Thank you for sharing your valuable time with us.

END RECORDING
